# Genetic diversity and association analysis between agronomic traits and EST-SSR markers in Chinese chive (*Allium tuberosum*)

**DOI:** 10.3389/fpls.2026.1785981

**Published:** 2026-03-23

**Authors:** Xiaojie Hou, Xingting Li, Youxiang Jiao, Xinjuan Guo, Dan Zhang, Yang Gao, Yuting Liu, Xiaojing Cheng, Sen Li

**Affiliations:** Shanxi Key Laboratory of Germplasm Resources Innovation and Utilization of Vegetable and Flower, College of Horticulture, Shanxi Agricultural University, Taigu, Shanxi, China

**Keywords:** *Allium tuberosum*, Anthocyanin content, association analysis, EST-SSR, flavonoid biosynthesis, genetic diversity, population structure, transcriptome

## Abstract

Chinese chive (*Allium tuberosum*) is an economically important vegetable and medicinal crop, yet research on its genetic diversity and population structure remains limited. In this study, we identified 68,248 SSR loci from chive transcriptome data and developed a set of corresponding SSR markers. After validation and screening, 59 polymorphic SSR markers were used to genotype 82 chive germplasm accessions. Clustering analysis and population structure d analysis assigned these accessions into three distinct groups. We conducted principal component analysis, correlation analysis, and marker-trait association analysis with FDR correction for 18 agronomic traits using GLM (Q) and MLM (Q+K) models. At the P ≤ 0.05 significance level, 2 and 30 significant trait-associated markers were identified by GLM and MLM, respectively. The marker sxauAt427 was significantly associated with anthocyanin content in both models, and its linked gene Unigene-84067 participates in the flavonoid biosynthesis pathway, which supplies critical precursors for anthocyanin production. Expression analysis further showed that key flavonoid biosynthesis genes were significantly upregulated in purple-rooted chives compared with white-rooted chives, providing a molecular basis for the root color difference. These results offer valuable genetic resources and theoretical support for gene mining, functional dissection of agronomic traits, and molecular breeding in Chinese chive.

## 1 Introduction

Chinese chives (*Allium tuberosum*) are an (1) important perennial herbaceous vegetable in China with a long history of cultivation and consumption. Native to China, they subsequently spread throughout East Asia and then to regions worldwide, developing a rich diversity of local varieties through long-term natural and artificial selection. The sulfur compounds abundant in Chinese chives confer a unique flavor (Liu et al., 2021), while modern nutritional and pharmacological research has also revealed broader functional properties of this crop (Moon et al., 2021). Studies indicate that Chinese chives contain antioxidant and anti-inflammatory compounds, exhibit antibacterial effects, and demonstrate anti-diabetic and hepatoprotective actions; these beneficial characteristics are primarily attributed to their organic sulfur compounds, polyphenols, and saponins (Tang et al., 2017; Ni et al., 2019; Putnik et al., 2019).

A typical characteristic of purple-rooted Chinese chives is the purple or purplish-red coloration at the base of their pseudostems (leaf sheaths). This trait is primarily attributed to their high anthocyanin content, particularly the accumulation of cyanidin derivatives. Anthocyanins play irreplaceable roles in enhancing plant resistance, maintaining human health, and food coloring, while also extending the shelf life of agricultural products (Qu et al., 2021; Sun et al., 2023). For humans, research evidence indicates that anthocyanins may help prevent cancer, stroke, and neurological diseases (Xu et al., 2014; Karanjalker et al., 2018). Therefore, purple-rooted Chinese chives not only possess a distinctive color but are also generally considered to have superior nutritional value compared to common white-rooted Chinese chives. The advantage of white-rooted Chinese chives lies in their tender texture and low fiber content (Medhia et al., 2022). The phenotypic differences between them may be related to genetic variations in anthocyanin biosynthesis-related genes and their regulatory networks. Understanding these genetic mechanisms has become a core task in breeding efforts aimed at synergistically improving appearance, flavor, and nutritional quality.

The genetic inheritance of traits in Chinese chives is highly complex, which makes the screening of progeny derived from sexual reproduction based on phenotypic traits extremely challenging. This approach is not only time-consuming but also low in accuracy, two drawbacks that are highly unfavorable for germplasm improvement and new variety breeding of Chinese chives. To solve this problem in Chinese chive breeding, molecular markers have been increasingly applied in genetic research. Among them, SSR molecular markers have become central tools for revealing genetic patterns and achieving efficient selection of desirable traits. This is primarily owing to their advantages of codominant inheritance, high polymorphism, and strong repeatability (Li et al., 2020a). However, the application of SSR markers in Chinese chives faces significant obstacles due to the crop’s genomic characteristics. Chinese chives are a tetraploid plant (2n = 4× = 32) with an estimated genome size of approximately 31.2 Gb (Baranyi and Greilhuber, 1999), which poses great challenges to the direct development of genomic SSR markers (Cheng et al., 2013).

Given the difficulty in developing genomic SSR markers for Chinese chives, transcriptome-derived SSR markers are the core of this study. EST-SSRs are derived from transcribed regions of the genome, which are highly conserved and closely associated with functional genes, making them ideal for genetic diversity analysis and trait association studies in crops with complex genomes. Despite their advantages, association analysis based on EST-SSRs has inherent limitations when applied to clonally propagated, highly heterozygous autotetraploid crops such as Chinese chives. Specifically, clonal propagation lacks genetic recombination events during reproduction, effectively maintaining high heterozygosity in Chinese chive populations. Additionally, the autotetraploid nature of Chinese chives exacerbates the complexity of allelic diversity and dosage effects. This often leads to indistinct bands and multiple peaks during SSR genotyping, thereby reducing the accuracy of genotypic data. Furthermore, due to the large and complex genome of Chinese chives, the number of developable EST-SSR markers is limited and their density is low, resulting in insufficient genome coverage. This deficiency increases the risk of missing key associated loci for complex agronomic traits (Fickett, 2018).

Molecular marker technology has been widely used in plant genetic research, but its application in Chinese chive research remains limited. Among various molecular markers, ISSR and RAPD markers are the earliest types attempted in Chinese chive research. To date, only a few studies have reported the applications of ISSR and RAPD markers in Chinese chives (Pan et al., 2005; Li et al., 2009; Zhang et al., 2012; Li et al., 2012). However, these two types of markers have inherent limitations such as poor repeatability and dominant inheritance, which further restrict their wide application in high-precision genetic research and breeding of Chinese chives. With the advancement of transcriptome sequencing technology, EST-SSR markers have gradually replaced traditional dominant markers and become the preferred tool for crop genetic research, thanks to their high stability and close association with functional genes, thus addressing the aforementioned limitations. Nevertheless, EST-SSR markers have also not been widely applied to the genetic research of Chinese chives.

Some researchers have carried out the development and screening of SSR loci based on the full-length transcriptome data of Chinese chives, successfully obtaining 39 pairs of polymorphic SSR markers and using these markers to complete the identification and analysis of 24 Chinese chive germplasm accessions (Li et al., 2020a). This study has laid an important methodological foundation for the development of Chinese chive SSR markers, but its application value is limited. Specifically, the limited number of screened polymorphic markers and the small sample size of the tested germplasm accessions make these markers unable to support subsequent studies on genetic diversity analysis and marker-assisted breeding of Chinese chives, further hindering the wide application of SSR markers in Chinese chive research.

In view of the above research status and limitations, this study expands the scale of SSR primer synthesis and screening to address these deficiencies and promote the wide application of SSR markers in Chinese chives research. Specifically, 500 pairs of SSR primers were synthesized in batches, and multiple rounds of rigorous screening were performed in combination with the genetic characteristics of autotetraploid Chinese chives, ultimately yielding 59 pairs of SSR markers with stable amplification and clear bands. Meanwhile, genetic diversity analysis and population structure analysis were conducted on 82 Chinese chive germplasm accessions, and association analysis was performed between the developed SSR markers and 18 agronomic traits. The results of this study provide an important theoretical basis for subsequent marker-assisted selection breeding of Chinese chives.

## 2 Materials and methods

### 2.1 Materials

A total of 82 Chinese chive germplasm resources were included in this study, including commercial varieties, local landraces, and wild-collected germplasm sourced from 28 distinct regions. Among these, 12 accessions were provided by the National Infrastructure for Vegetable Crop Germplasm Resources, National Mid-term Genebank for Vegetables (Beijing, China). Detailed information on the 82 germplasm accessions is provided in **Supplementary Table 1**. All materials were cultivated in the Chinese chive resource nursery of the Horticultural Station, Shanxi Agricultural University (Taigu District, Shanxi Province, China). Each variety was planted in 3 rows with a plant spacing of 15 cm and row spacing of 30 cm. All test plants had been established in the nursery for 2 years, and the field materials were uniformly managed according to standard agronomic practices for local Chinese chives.

### 2.2 Determination and statistical analysis of agronomic traits

At the initial harvest stage, (defined as the date when 30% of the plants reached the commercial standard and are ready for harvest), 3 plants with consistent growth were randomly selected to investigate agronomic traits. The investigated traits included plant height (PH; cm), plant width (PW; cm), leaf length (LL; cm), leaf width (LW; cm), pseudostem length above ground (PL; cm), pseudostem diameter (PD; mm), number of leaves (NL), leaf color (LC), leaf shape (LS), leaf apex (LA), leaf posture (LP), pseudostem color (PC), root color (RC), root color length (RCL; cm), and anthocyanin content (AC; mg/100g). Agronomic traits were also investigated at the initial bolting stage (defined as the date when 30% of the plants bolt), including flower shape (FS), flower stalk structure (FSS), and flower stalk length (FSL; cm). The trait descriptions are provided in **Supplementary Table 2**.

In this study, the measurement of Chinese chive agronomic traits strictly adhered to the standards specified in the *Descriptive Standards for Chinese Chive Germplasm Resources* published by the National Crop Science Data Center to ensure the standardization and comparability of phenotypic data. For qualitative traits (e.g., LC, RC), direct numerical calculations are not feasible. Therefore, different types of qualitative traits were assigned values prior to statistical analysis, laying a data foundation for subsequent multivariate statistical analysis.

Plants of similar size were selected for trait determination, with three biological replicates set for each trait. Prior to trait determination, equipment calibration was performed, and standardized operating procedures were followed to maintain consistent experimental conditions. This approach effectively reduced errors in material collection and phenotypic trait measurement.

The anthocyanin content in Chinese chive roots was determined using the pH differential method. This method only quantifies the total monomeric anthocyanin content in the sample and cannot distinguish specific anthocyanin monomer species. The identification of specific components requires further HPLC analysis (Tan et al., 2024).

Basic statistical indicators, including mean, standard deviation, Shannon-Wiener index, and coefficient of variation, were calculated using Excel 2019 software. Considering that Chinese chive agronomic traits include both qualitative and quantitative traits, mixed principal component analysis was performed using the PCAmixdata R package and Origin software (Chavent et al., 2022). Additionally, Origin 2024 software was employed to correlation analyses among 18 agronomic traits across 82 Chinese chive germplasm resources.

### 2.3 Transcriptome assembly, SSR identification and EST-SSR genotyping

Two sets of transcriptome data were used in this study to ensure the comprehensiveness and reliability of SSR marker development, and both were subjected to consistent quality control, filtering and assembly procedures to guarantee data comparability.

The first set was obtained from NCBI database (PRJNA673978), which included six tissue types of Chinese chive: root, stem, leaf, flower, inflorescence, and seed. Raw data were filtered using the Trimmomatic tool to obtain clean reads, which were subsequently assembled into *de novo* transcripts via the Trinity program (Grabherr et al., 2011; Bolger et al., 2014). These transcripts covered samples from different organs of Chinese chive, maximizing the coverage of SSR loci in the Chinese chive genome. This provides a broader sequence basis for the preliminary mining and development of subsequent SSR markers, ensuring that the obtained candidate SSR markers have stronger universality.

The second set of transcriptome data was newly generated in this study, aiming to support the investigation of intrinsic molecular differences between purple-root (PR) and white-root (WR) Chinese chives (corresponding to germplasm with high and low anthocyanin contents, respectively). Root samples of PR and WR Chinese chives were collected at the initial harvesting stage, with three biological replicates set for each type; each biological replicate was derived from a different independent plant to avoid individual bias. Transcriptome sequencing was performed on these samples, and new transcript sequences were obtained after sequencing and assembly. These new transcript sequences were used for homologous sequence alignment and screening of SSR markers developed from public NCBI data. Finally, SSR markers with corresponding homologous sequences were obtained, confirming reliable molecular marker resources applicable to subsequent experiments. This ensures the rigor of data application and the reliability of analysis results. Both sets of raw data were subjected to quality control and filtering using identical Trimmomatic parameters, and transcriptome *de novo* assembly was performed using the same Trinity software with consistent parameters. This consistency in data processing ensures the comparability of the two sets of transcriptome data. Furthermore, functional prediction of unigenes from the newly assembled transcripts was conducted by searching seven databases: COG, GO, KEGG, KOG, Nr, Nt, and Swiss-Prot databases.

In this study, Krait software was used to mine and analyze SSR loci from the newly assembled transcript sequences. The screening criteria were set as follows: mononucleotide repeats ≥ 10, dinucleotide repeats ≥ 6, and tri− to hexanucleotide repeats ≥ 5. No perfect−match filtering parameters were applied, and a total of 68,248 SSR loci were identified. Based on these SSR motifs, 30,041 primer pairs were successfully designed with the following parameters: length 18–27 bp, GC content 40%–80%, melting temperature (Tm) 58–65 °C with a Tm difference ≤ 2 °C between forward and reverse primers, and amplicon size 100–300 bp. In silico PCR was employed to filter out primers that targeted multiple specific loci (Kalendar et al., 2017). According to the obtained information, primer sequences were synthesized in batches by Tsingke Biotechnology Co., Ltd. (Beijing, China).

The specific procedures for DNA extraction and PCR amplification are as follows: Healthy Chinese chive leaves were collected, and DNA was extracted from fresh leaves using the CTAB method (Doyle and Doyle, 1987). Touch-down PCR amplification was performed as follows: pre-denaturation at 94 °C for 5 min, followed by 10 touch-down cycles (annealing temperature decreased by 1 °C per cycle) and 25 regular cycles, totaling 35 cycles. A final extension was carried out at 72 °C for 10 min, and the amplification products were stored at 4 °C. PCR products were detected by 9% polyacrylamide gel electrophoresis using 0.5×TBE buffer (Azhaguvel et al., 2009). Electrophoresis was conducted at a constant current of 340 mA with 2 electrophoresis boxes and 4 gels in total. This experimental setup effectively controlled heat generation, prevented band distortion, and ensured uniform fragment separation. Silver nitrate staining was applied after electrophoresis to visualize the bands.

In addition, to verify the reliability of the newly generated transcriptome data (the second set) in this study, we performed RT-qPCR verification experiments. Eight genes were selected for verification, including Unigene-84067 and seven additional DEGs enriched in the plant flavonoid biosynthesis pathway that correlated with Unigene-84067 expression (gene names and annotation details are listed in **Table 1**). Primer information for qPCR is shown in **Supplementary Table 3**. Total RNA was extracted using the Vazyme RC401–01 RNA extraction kit, and cDNA was synthesized with the TransGen AU341 cDNA synthesis kit. After screening, the stably expressed GAPDH gene (glyceraldehyde-3-phosphate dehydrogenase) was selected as the internal reference gene, with the forward primer sequence CCAACTGCTTAGCCCCTCTT and the reverse primer sequence CCGTCAACAGTCTTCTGGGT. The PCR conditions for RT-qPCR were set as follows: pre-denaturation at 94 °C for 30 s; followed by 40–45 amplification cycles, each consisting of annealing at 50–60 °C for 15 s and extension at 72 °C for 10 s; after the PCR program, melting curve analysis was performed to verify the specificity of the amplified products; the relative expression levels of the target genes were calculated using the 2−ΔΔCt method, and all figures were generated using GraphPad Prism 8 software.

**Table 1:** Information of 8 DEGs used for RT-qPCR.

Gene-ID	Gene symbol	Description
Unigene-.60844	CYP73A-1	trans-cinnamate 4-monooxygenase [EC:1.14.14.91]
Unigene-.57267	CYP73A-2	trans-cinnamate 4-monooxygenase [EC:1.14.14.91]
Unigene-.64377	HCT	shikimate O-hydroxycinnamoyltransferase [EC:2.3.1.133]
Unigene-.84067	CHS	chalcone synthase [EC:2.3.1.74]
Unigene-.59386	caffeoyl-CoA O-methyltransferase-1	caffeoyl-CoA O-methyltransferase [EC:2.1.1.104]
Unigene-.60347	caffeoyl-CoA O-methyltransferase-2	caffeoyl-CoA O-methyltransferase [EC:2.1.1.104]
Unigene-.55724	chalcone isomerase	chalcone isomerase [EC:5.5.1.6]
Unigene-.38647	F3H	naringenin 3-dioxygenase [EC:1.14.11.9]

### 2.4 Genetic diversity and population structure analysis

For each primer pair, amplified bands from different genotypes were scored individually. Only clear, reproducible bands with clean background were scored. Bands that were faint, smeared, or too closely spaced to be reliably distinguished were not scored and treated as missing data. The size of amplified fragments was determined against a DNA ladder, and only bands within the expected molecular weight range were recorded. Those outside the range were discarded. Each unambiguous fragment was treated as an independent binary locus, with presence scored as “1”, absence as “0”, and unreadable loci as missing data (–9) (Example gel: **Supplementary Figure 1**). A 0/1 matrix was constructed using Excel 2019 (Huang et al., 2025). The number of observed alleles (Na), number of effective alleles (Ne), Shannon’s information index (I), gene diversity (H), and polymorphism information content (PIC) were calculated manually. Based on Nei’s genetic distance, cluster analysis was performed using the Unweighted Pair-Group Method with Arithmetic Mean (UPGMA), and a dendrogram was constructed using R packages.

Population genetic structure was analyzed using Structure 2.3.4 software based on a Bayesian clustering approach (Liu et al., 2020). The model assuming no prior population information (Pritchard et al., 2000), with the burn-in period set to 10,000 and the Markov chain Monte Carlo (MCMC) chain length to 100,000. The number of clusters (K) was set from 1 to 10, with 20 independent runs for each K. The optimal K value was calculated and determined via Structure Selector (Liu et al., 2024), and the Q values of each germplasm were processed and integrated using CLUMPP (Jakobsson and Rosenberg, 2007).

### 2.5 Population genetic structure and differentiation analysis

Based on the genotypic data of 59 EST-SSR markers, the pairwise genetic distances among samples were calculated using the binary Jaccard coefficient. This analysis was implemented using the vegan package in R software (Jaccard, 1901; Oksanen et al., 2022). Analysis of Molecular Variance (AMOVA) was used to assess the hierarchical distribution of genetic variation. A total of 999 permutation tests performed to evaluate the significance of variance components (Excoffier et al., 2018). Using the variance components derived from the AMOVA analysis, the overall and pairwise population genetic differentiation indices (Fst) were calculated following Wright’s Fst formula. This formula is suitable for the analytical requirements of dominant markers (Wright, 1951; Holsinger and Weir, 2009).

### 2.6 Association analysis of traits in 82 Chinese chives

TASEEL 5.0 software was used to analyze associations between SSR markers and agronomic traits in Chinese chives (Zheng et al., 2021). Two statistical models were employed: the General Linear Model (GLM, Q) and the Mixed Linear Model (MLM, Q+K). The first five principal components (PC = 5) were incorporated into both models as population structure covariates to correct for population stratification (Yu et al., 2006). The Q model incorporates population structure information to correct for the interference of population stratification on association results. On this basis, the MLM model further integrates kinship information (K matrix), which effectively reduces the probability of false positive associations. The kinship matrix (K matrix) required for the analysis was constructed using the rrBLUP program. This program precisely estimates kinship among germplasm resources based on SSR marker genotype data, providing critical parameter support for MLM model calculations (K matrix, see **Supplementary Table 4**). To strictly control false positives and ensure the robustness of the results, P ≤ 0.05 was used as the preliminary screening threshold. Subsequently, FDR (False Discovery Rate) correction for multiple testing (Jaiswal et al., 2012). This approach identified robust and reliable marker–trait associated loci, improving the accuracy and statistical reliability of the association analysis.

## 3 Results

### 3.1 Analysis of agronomic traits

This study analyzed 10 quantitative traits and 8 qualitative traits across 82 Chinese chive germplasm resources. Key statistical indicators, including mean, standard deviation, Shannon-Wiener index, and coefficient of variation, were calculated to evaluate genetic diversity and phenotypic variation. Detailed statistical data are provided in **Supplementary Table 5**. For quantitative traits, statistical results revealed Shannon-Wiener indices (approximately 4.40) for growth-related traits, including PH, LL, NL, and FSL. This indicates similar and relatively high genetic diversity among these traits. Additionally, these traits exhibited relatively low coefficients of variation (CV), reflecting limited phenotypic variation and strong genetic stability within the tested population. In contrast, RCL and AC showed notably lower Shannon-Wiener indices (3.73 and 2.61, respectively), indicating low genetic diversity. Their CVs were extremely high (118.94% and 289.25%), which highlights significant phenotypic differentiation and complex genetic variation patterns for these two traits. Among all germplasm, accession A14 displayed an extreme phenotype with extremely high anthocyanin content, serving as a typical example of AC variation.

For qualitative traits, PC and LP had Shannon-Wiener indices ranging from 4.26 to 4.38, indicating high genetic diversity. Regarding CV, LS, FS, FSS, and RC had values of 53.49%, 40.58%, 43.00%, and 64.33%, respectively. RC had the highest CV, suggesting significant phenotypic variation and rich genetic backgrounds for these qualitative traits in the tested population.

Overall, most quantitative and qualitative traits in the 82 Chinese chive germplasm resources showed high genetic diversity. This provides a rich gene pool for superior trait selection in chive breeding. Notably, specific traits (e.g., AC, RC) exhibited high variability, offering critical breakthrough points for targeted improvement of nutritional quality and appearance traits.

**Figure 1:**
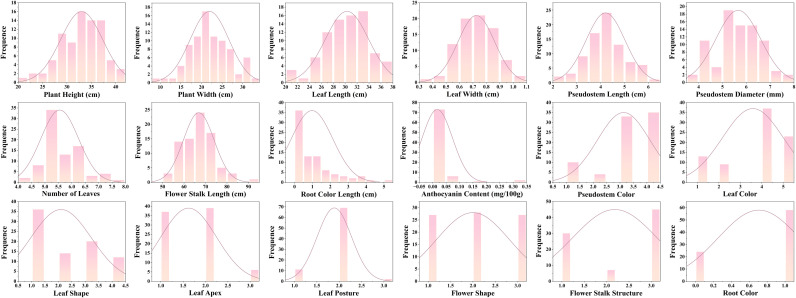
Frequency distribution diagrams of 18 agronomic traits in 82 Chinese chive germplasm resources.

Phenotypic frequency distribution analysis (**Figure 1**) showed that most quantitative traits approximated a normal distribution. Phenotypic variation within the population was continuous and concentrated around the mean, as demonstrated by PH, PW, LL, LW, PL, PD, NL, and FSL. These traits had distinct, symmetric peaks, indicating good phenotypic consistency and moderate variation amplitude in the tested population. A few quantitative traits exhibited skewed distributions. For example, RCL peaks were concentrated around 0, with very few individuals showing longer root color. Qualitative traits, by contrast, displayed multimodal or discrete frequency distributions with discontinuous phenotypic classification. For PC and LC, peaks were concentrated in specific value intervals but also appeared in other intervals. This indicates dominant color phenotypes alongside existing diversity. Traits including LS, LA FS, FSS and RC all show multimodal distributions, reflecting multiple typical phenotypes—a characteristic feature of qualitative traits.

### 3.2 Principal component analysis and correlation analysis

Principal component analysis was performed to evaluate the phenotypic variation structure of 18 agronomic traits across the 82 Chinese chive germplasm resources. Key results are summarized below.

**Figure 2:**
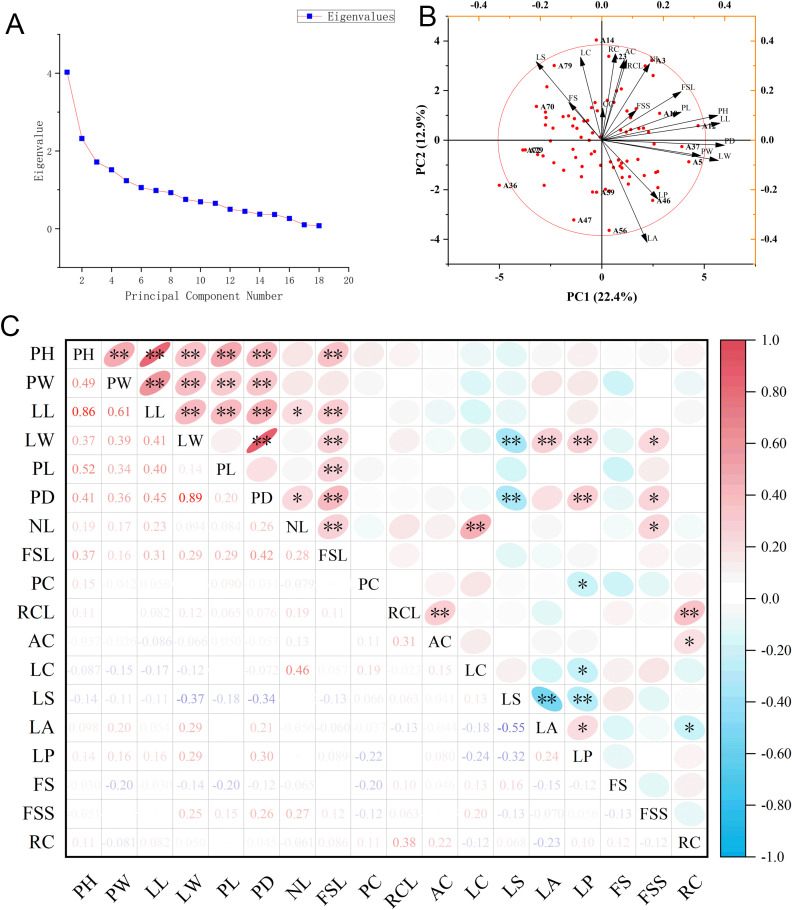
Principal component analysis plot and correlation heatmap of 18 agronomic traits in Chinese chives. **(A)** scree plot of principal component analysis (PCA); **(B)** PCA scatter plot; **(C)** correlation heatmap. "*" denotes a significant correlation (p < 0.05) and "**" denotes a highly significant correlation (p < 0.01).

The cumulative contribution rate of the first three principal components (PC1, PC2, PC3) reached 44.797%. Specifically, PC1, PC2, and PC3 contributed 22.40%, 12.90%, and 9.50%, respectively (**Supplementary Table 6**). These three principal components collectively captured the primary structural characteristics of phenotypic variation within the tested germplasm population. PC1 was dominated by vegetative growth-related traits. High positive loadings were observed for PW (0.399), LW (0.379), PH (0.378), and LL (0.385). These traits collectively determine the overall scale of vegetative growth in Chinese chives. Germplasm resources with high PC1 scores (e.g., A5, A12, A19) typically exhibited tall plant architecture, thick pseudostems, and broad, elongated leaves. This phenotypic characteristic indicates excellent vegetative growth potential, making these germplasms important candidate parents for high-yield breeding. PC2 primarily reflected leaf-related traits. Significant positive loadings were detected for LC (0.335), NL (0.309), and LS (0.317). In contrast, Leaf Apex (LA, -0.413) showed a significant negative loading.This loading pattern indicates synergistic variation among leaf number, leaf morphology, and leaf tip characteristics. Germplasm with high PC2 scores (e.g., A14, A3, A11) featured dense leaf arrangement and regular leaf shape. Such germplasms are well-suited for morphological improvement in leaf-type Chinese chive breeding. PC3 was mainly associated with reproductive growth traits. FMS (0.459) exhibited a significantly higher loading than other traits in this component. This result suggests that the morphological differentiation of flower stalks constitutes the third major dimension of phenotypic variation in the tested population. It also provides a phenotypic basis for selecting flower stalk-specialized Chinese chive varieties. The biplot of germplasm accessions and trait vectors is presented in **Figures 2B**, visually illustrating the relationships between germplasm resources and key agronomic traits.

Correlation analysis was conducted to explore the interdependencies among the 18 agronomic traits. Detailed correlation results are shown in **Figures 2C**, with key findings summarized as follows. Correlation analysis of quantitative traits revealed significant synergistic associations among core vegetative growth-related traits. PH, PW, LL, and LW showed extremely significant positive correlations with each other. This indicates a highly coordinated vegetative growth pattern in Chinese chives: taller plant types are typically accompanied by wider plant widths, longer leaves, and broader leaves. Further analysis showed that PH, PW, and LL all exhibited extremely significant positive correlations with both PL and PD. This reflects strong interdependence in the growth and development of above-ground vegetative organs (leaves and pseudostem), indicating synergistic nutrient allocation among these organs. FSL showed extremely significant positive correlations with PH, LL, LW, PL, PD, and NL. This result demonstrates that vigorous vegetative growth significantly promotes flower stalk development, highlighting the close link between vegetative and reproductive growth in Chinese chives. Among root color-related quantitative traits, RCL showed a significant positive correlation with AC. This positive association indicates that the extension of root coloration is closely related to anthocyanin accumulation, which is consistent with the anthocyanin-enriched phenotype of purple-root Chinese chives.

Correlation analysis of qualitative traits revealed clear genetic correlations among multiple traits. RC showed an extremely significant positive correlation with RCL and a significant positive correlation with AC. This implies that germplasms with darker root colors (e.g., purple roots) usually have longer coloring lengths and higher anthocyanin accumulation. Meanwhile, RC exhibited a significant negative correlation with LC, suggesting that root coloring characteristics and leaf color traits may be regulated by genetically correlated mechanisms. Correlations were also observed among leaf-related qualitative traits. LP showed a significant positive correlation with LA morphology and an extremely significant positive correlation with LS. This indicates that the overall growth posture of Chinese chive leaves exhibits synergistic differentiation patterns with leaf apex and overall leaf morphology, reflecting the genetic linkage of traits involved in leaf morphogenesis.

### 3.3 Transcriptome assembly, microsatellite identification, and EST-SSR genotyping

**Figure 3:**
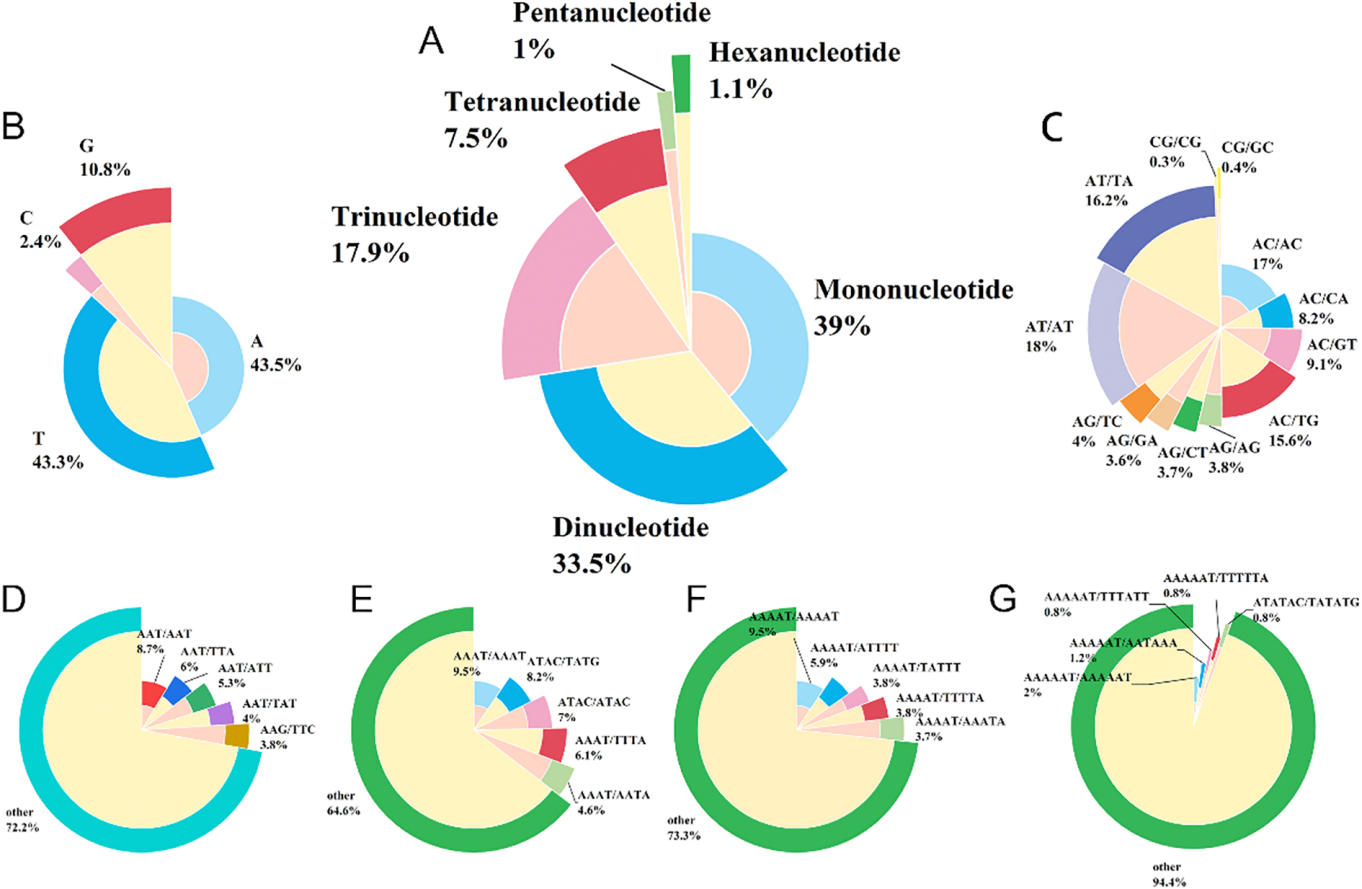
Statistical distribution chart of SSR loci in the transcriptome of Chinese chives. **(A)** the proportion of nucleotide types; **(B)** the proportion of mononucleotides; **(C)** the proportion of dinucleotides; **(D)** the proportion of trinucleotides; **(E)** the proportion of hexanucleotides; **(F)** the proportion of pentanucleotide; **(G)** the proportion of hexanucleotide.

Transcriptome assembly of NCBI-derived Chinese chive sequencing data yielded high-quality clean reads after strict quality control. *De novo* assembly generated a final transcriptome dataset of 512.72 MB, which provided a reliable sequence foundation for subsequent SSR molecular marker development. SSR molecular markers were identified based on the assembled transcript data, with all detected SSR loci (including perfect, imperfect, and compound SSRs) included in the analysis to ensure result comprehensiveness. A total of 68,248 SSR loci were identified, covering 6 repeat types from mononucleotide to hexanucleotide repeats (**Figure 3**). The number and proportion of each repeat type differed significantly: Mononucleotide repeats were the most dominant type, with 26,631 loci accounting for 39.02%; dinucleotide repeats ranked second, totaling 22,842 loci (33.47%); trinucleotide repeats contained 12,210 loci (17.89%); tetranucleotide repeats had 5,103 loci (7.48%); pentanucleotide and hexanucleotide repeats were rare, with 709 (1.04%) and 753 (1.10%) loci respectively. Overall, mononucleotide and dinucleotide repeats collectively accounted for 72.49% of all SSR loci, while tetra- to hexanucleotide repeats contributed only 9.62%. This distribution pattern indicated that Chinese chive SSR loci were predominantly composed of short repeat motifs.

Base composition and dominant motifs varied significantly among different repeat types, with distinct characteristics for each type:

Mononucleotide repeats included four base repeat types (A, T, C, G), and exhibited a clear AT preference. A and T repeats accounted for 43.50% (11,584 loci) and 43.29% (11,529 loci) of mononucleotide repeats respectively, with a cumulative proportion exceeding 86%. In contrast, G and C repeats were relatively scarce, accounting for 10.77% (2,867 loci) and 2.44% (650 loci) respectively. Twelve base combination types were detected in dinucleotide repeats, with AT/AT as the dominant motif (4,113 loci, 18.01% of dinucleotide repeats). CG/CG was extremely rare, with only 71 loci (0.31%), consistent with the AT preference observed in mononucleotide repeats. Trinucleotide repeats showed high motif diversity, with 207 identified types, and AAT/ATT-related motifs were predominant. The top five dominant motifs (AAT/AAT, AAT/TTA, AAT/ATT, AAT/TAT, AAG/TTC) accounted for 27.83% of trinucleotide loci, while the remaining 202 types contributed 72.17%. Tetranucleotide repeats also contained 207 motif types, with AAAT-related motifs as the main type. The five most abundant motifs (AAAT/AAAT, TAC/TATG, ATAC/ATAC, AAAT/TTTA, AAAT/AATA) accumulated to 35.40% of tetranucleotide loci, and the remaining 202 types accounted for 64.60%. Pentanucleotide and hexanucleotide repeats exhibited the highest motif richness, with 225 and 497 types detected respectively. However, their total number was small, and no obvious dominant motifs were formed, which may be related to their low abundance in the transcriptome.

A total of 30,041 primer pairs were designed based on the identified SSR loci. After screening for specificity and validation, 524 primer pairs were randomly selected for batch synthesis and amplification band detection.

Finally, 59 highly polymorphic primers were selected based on clear amplification bands and correct product sizes (detailed primer information in **Supplementary Table 7**). These primers were used for subsequent germplasm genotyping and marker-trait association analysis, providing reliable molecular tools for related research.

### 3.4 Genetic diversity and population structure analysis

Genetic diversity indices were calculated based on the genotyping data of 59 SSR markers, and key results are summarized below. A total of 396 alleles (Na) were detected, with an average of 6.7 alleles amplified per marker (**Supplementary Table 8**). The number of effective alleles (Ne) varied from 1.309 to 5.107, with an average value of 3.305. Nei’s genetic diversity index (H) ranged between 0.315 to 1.072, with a mean value of 0.877. The Shannon’s information index (I) spanned 0.399 to 1.685, averaging 1.225, while the polymorphism information content (PIC) ranged from 0.208 to 0.775. According to polymorphism classification criteria, 78% of the primers exhibited a high level of polymorphism. The amplicon sizes of the 59 SSR markers ranged from 100 to 400 bp. These results collectively indicate that the tested Chinese chive germplasm resources possess high genetic diversity and polymorphism, providing a solid material basis and marker support for subsequent trait association analysis.

**Figure 4:**
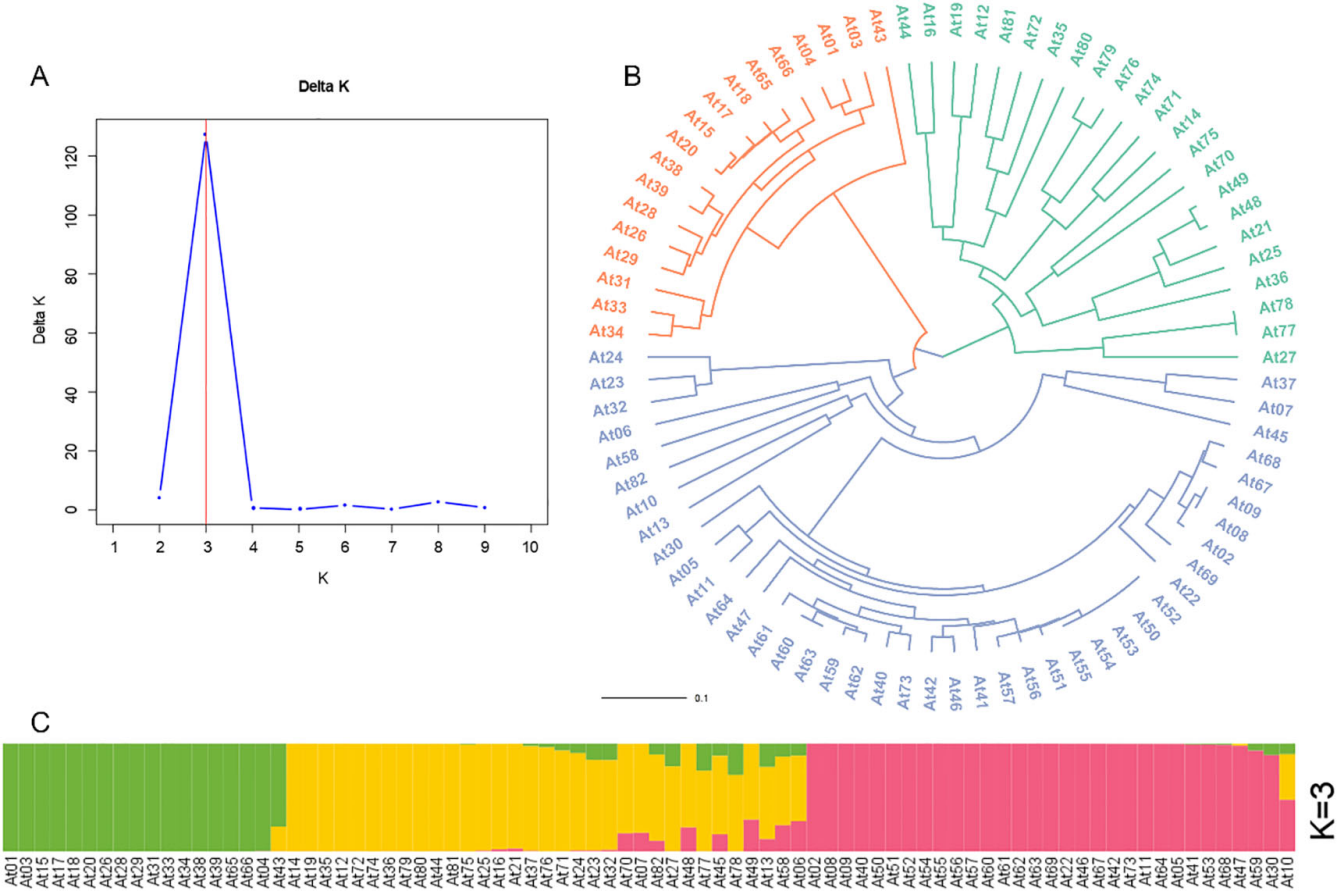
Structure analysis of 82 Chinese chive germplasm resources. **(A)** The relationship between K-value and ΔK value. **(B)** dendrogram of 82 chives germplasm resources constructed by the UPGMA. **(C)** Clustering of 82 Chinese chive germplasm resources.

Cluster analysis was performed on 82 germplasm resources to clarify their genetic relationships. The results (**Figure 4B**) showed that all resources were clearly divided into three clusters (Group I, Group II, and Group III). Group I contained 41 resources (marked in blue), Group II included 23 resources (marked in green), Group III, including 18 resources (marked in red).

Population structure analysis was also conducted to further explore the genetic composition of the 82 germplasm resources. The optimal number of population divisions was determined by calculating ΔK values and a peak ΔK value was observed when the assumed population number K = 3 (**Figure 4A**). (**Figure 4A**). This indicates that the 82 germplasm resources can be divided into three independent subpopulations (Pop I, Pop II, Pop III), which was highly consistent with the results of the cluster analysis. Detailed characteristics of each population are as follows (**Figure 4C**):

Pop I (marked in pink) includes 31 resources, all derived from Group I of the cluster analysis. This population showed extensive phenotypic variation, with significant differences among individual germplasm resources. Pop II (marked in yellow) contained 33 resources, with relatively uniform phenotypes. The average values of some growth indicators in this population were slightly lower than those in the other two subpopulations. Among these 33 resources, 11 were from Group I and 22 from Group II of the cluster analysis. Pop III (marked in green) comprised, which completely covered all individuals of Group III in the cluster analysis. This population exhibited high phenotypic consistency, characterized by vigorous vegetative growth and strong bolting ability.

### 3.5 Population genetic structure and differentiation analysis

To clarify the distribution pattern of genetic variation and the degree of differentiation among the three Chinese chive populations, genetic differentiation analysis was performed based on the genotyping data of 59 EST-SSR markers. Key analyses included analysis of molecular variance (AMOVA) and inter-population genetic differentiation index (Fst) assessment.

AMOVA results showed that genetic variation was distributed both among and within subpopulations. Of the total genetic variation, 53.29% was attributed to inter-population differentiation (variance component = 0.0922, P < 0.001), while 46.71% originated from intra-population differences (variance component = 0.0808). This finding indicates that genetic differentiation among the three Chinese chive populations reached an extremely significant level (P < 0.001). Notably, inter-population genetic differences were greater than intra-population differences, demonstrating that the three subpopulations have formed distinct genetic structures.

Fst analysis further quantified the degree of genetic differentiation among subpopulations. The overall genetic differentiation index (Fst), derived from AMOVA variance components, was 0.533. This value indicated high genetic differentiation among the three subpopulations, as Fst > 0.25 is generally recognized as a threshold for high differentiation. Pairwise inter-population Fst analysis revealed clear differences in differentiation degrees among different population combinations. The highest differentiation was observed between Pop1 and Pop3 (Fst = 0.858), followed by Pop2 and Pop3 (Fst = 0.499). In contrast, the differentiation between Pop1 and Pop2 was relatively lower (Fst = 0.401). All pairwise Fst values exceeded the threshold for high differentiation, which was consistent with the AMOVA results and further confirmed significant genetic differentiation among the three subpopulations.

In conclusion, the three Chinese chive subpopulations have formed distinct genetic structures with extremely high inter-population genetic differentiation. This significant differentiation may be closely related to factors such as long-term geographic isolation, artificial selection, genetic drift, or other related factors.

### 3.6 Association analysis of agronomic traits among 82 Chinese chives

Marker-trait association analysis was performed to identify SSR markers significantly associated with Chinese chive agronomic traits, using two statistical models for cross-validation. Dual-model validation ensured the reliability of association signals, ultimately leading to the identification of a set of SSRs closely linked to multiple agronomic traits. Core results are summarized below.

At the significance level of P ≤ 0.05, with false positive control and model fitting evaluation conducted to ensure result reliability. Significant differences were observed between the two models in detecting marker-trait associations (detailed results are provided in **Supplementary Tables 9, 10**). The GLM detected only 2 significantly associated markers, each corresponding one agronomic trait. Specifically, the two markers were associated with RCL and AC, respectively. In contrast, the MLM detected a total of 30 significantly associated markers, with substantial variation in the number of markers associated with different traits. LP had the largest number of associated markers (8), followed by LA (7), FSS (5), LW (4), LC (3), NL (2), and AC (1). The QQ plots and Manhattan plots were used to visualize the association results of each trait under both models, with detailed figures provided in **Supplementary Figure 2**.

**Figure 5:**
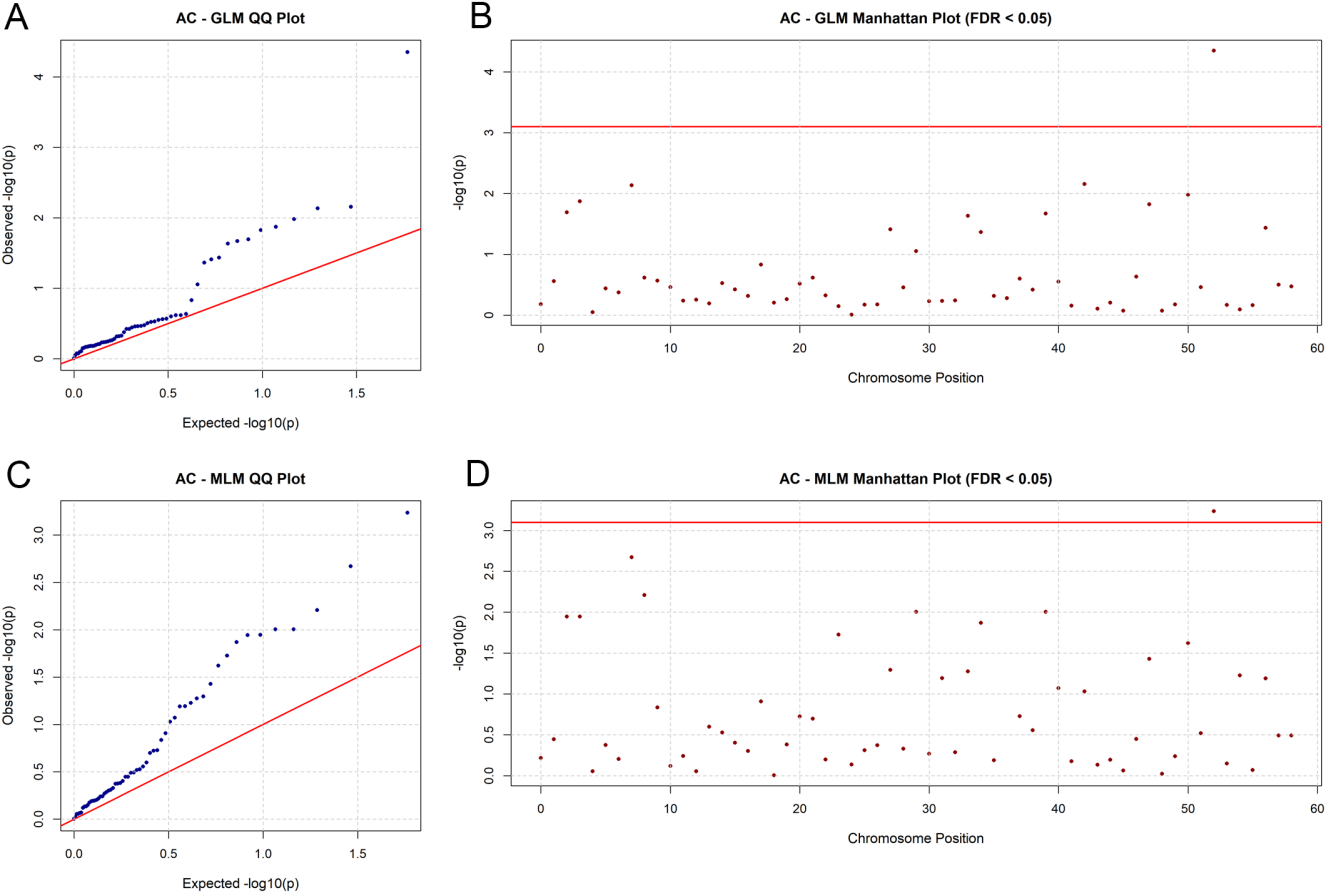
Association analysis results for agronomic trait AC in Chinese chive. **(A)** Quantile-quantile (QQ) plot under the general linear model (GLM); **(B)** Manhattan plot under the general linear model (GLM); **(C)** Quantile-quantile (QQ) plot under the mixed linear model (MLM); **(D)** Manhattan plot under the mixed linear model (MLM), with the red horizontal line indicating the significance threshold of FDR < 0.05.

Among all association markers, sxauAt427 showed the most prominent biological significance. This marker was significantly associated with total anthocyanin content (AC) in both models (**Figure 5**). Additionally, sxauAt427 was significantly correlated with Unigene-84067 (HCT), which encodes the HCT enzyme involved in the flavonoid biosynthesis pathway. As a key enzyme in the phenylpropanoid pathway, HCT acts upstream of anthocyanin synthesis and provides key precursors for total anthocyanin biosynthesis. This finding clarifies the molecular regulatory basis underlying the differences in anthocyanin content among Chinese chive germplasm resources. It should be noted that only total monomeric anthocyanin content was determined in this study; the identification of specific anthocyanin components requires further investigation.

### 3.7 Transcriptome analysis

To explore the intrinsic molecular differences between purple-root (PR) and white-root (WR) Chinese chive, we collected root samples from germplasm with high (PR) and low (WR) anthocyanin contents at the initial harvesting stage. Three biological replicates were set for each group, with each replicate derived from a distinct independent plant. Two sets of transcriptome data were used in this study, with consistent 150-bp paired-end sequencing specifications but different sequencing platforms. The newly generated transcriptome data in this study were sequenced on the DNBSEQ−T7 platform, while the public transcriptome data (PRJNA673978) were obtained from the Illumina HiSeq 4000 platform.

Raw sequencing data were filtered to obtain clean reads, and detailed filtering parameters and clean read counts are presented in **Supplementary Table 11**. Since only sequence homology alignment was conducted without gene expression quantification, the difference in sequencing platforms did not affect the reliability of the results. *De novo* assembly of the newly generated transcriptome data produced 400,850 transcripts and 147,528 unigenes, with good assembly quality. Key assembly metrics were as follows: transcript N50 of 1,431 bp, mean transcript length of 1,063 bp; unigene N50 of 1,060 bp, mean unigene length of 852 bp; average GC content of 42.15%; and average read−mapping rate of 74.66% (ranging from 72.75% to 78.78%). The newly assembled transcript sequences were used for homologous sequence alignment and screening of SSR markers developed from public transcriptome data.

Previous association analysis identified SSR markers significantly associated with anthocyanin content, but the specific functions of these associated genes remain unclear due to the lack of a reference genome for Chinese chives. Thus, PR and WR Chinese chives were selected for transcriptome sequencing to clarify the molecular mechanisms underlying anthocyanin content differences. Pearson correlation coefficient analysis, with three biological replicates for both PR and WR groups, showed strong intra-group sample correlations. This indicated high similarity in gene expression patterns among replicates of the same group. In contrast, inter−group correlations were markedly lower and consistently below intra−group levels, reflecting high intra-group similarity, clear inter-group separation, and a distinct association with phenotypic differences between the two groups (**Supplementary Figure S3**). Further sample cluster analysis confirmed that biological replicates of WR and PR groups clustered into separate branches. This verification confirmed the consistency of intra−group gene expression patterns and the reliability of the experimental replicates. Heatmap visualization showed distinct color separation between the two groups, with many genes displaying opposite expression trends. These observations provided direct evidence for substantial differences in the overall transcriptional profiles of PR and WR Chinese chives (**Supplementary Figure S4**). Principal component analysis (PCA) was conducted based on the FPKM values of all samples to further characterize transcriptome differences. Low-expression genes were filtered out first, retaining genes with FPKM ≥ 1 in at least 3 of the 6 samples, resulting in 64,056 genes for subsequent analysis. PCA results showed that the first principal component (PC1) and second principal component (PC2) clearly distinguished PR and WR Chinese chive samples (**Supplementary Figure S5**). The two groups exhibited a significant separation trend along PC1 with no overlap, indicating substantial global transcriptome differences between PR and WR Chinese chives and providing solid data support for subsequent key gene screening.

**Figure 6:**
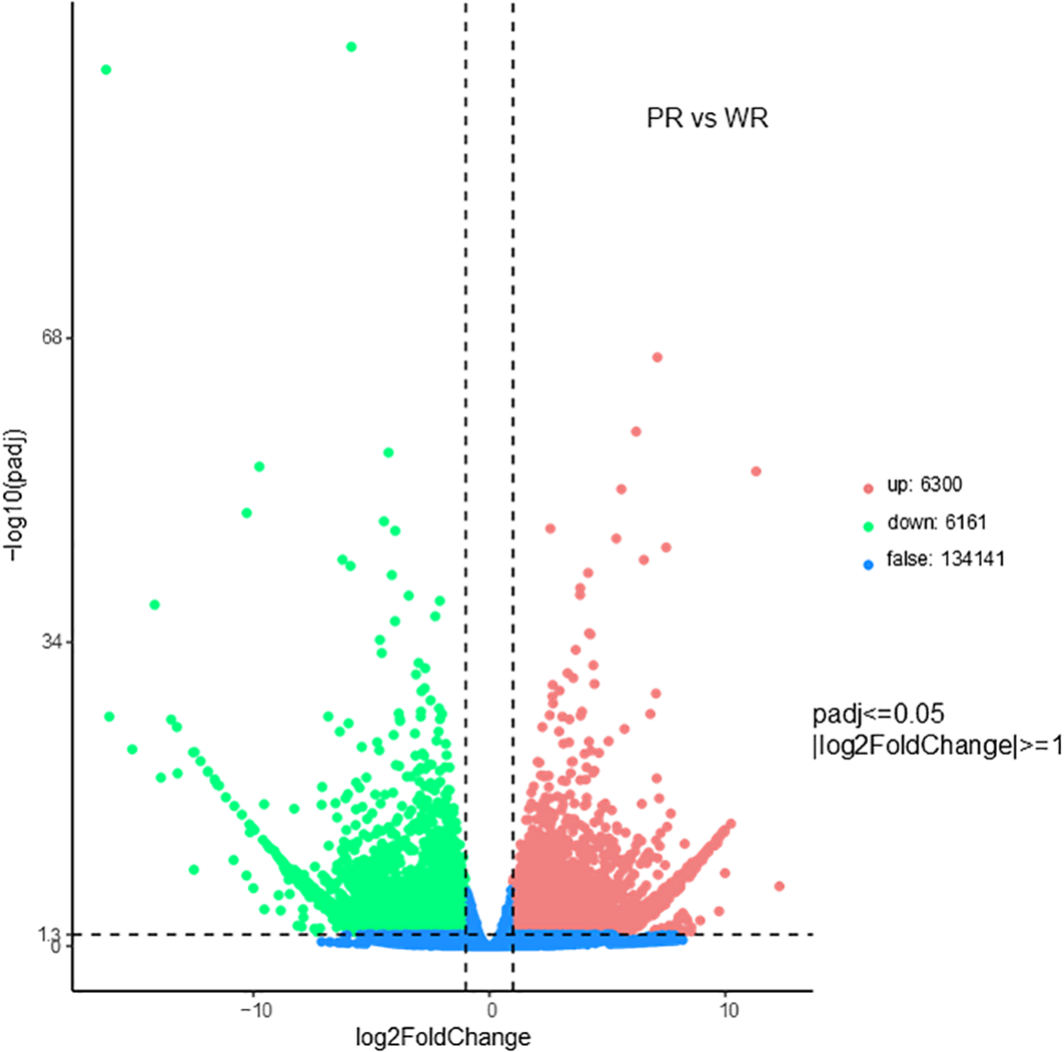
Volcano plot of transcriptomic differentially expressed genes between PR and WR.

Differentially expressed gene (DEG) analysis identified 12,460 significant DEGs in total, including 6,300 up−regulated genes (50.6%) and 6,160 down−regulated genes (49.4%). This distribution indicated a relatively balanced pattern of up- and down-regulation among DEGs, and the corresponding volcano plot is shown in **Figure 6**. A total of 147,528 unigenes were obtained in this study, and functional annotation results demonstrated good integrity and reliability of the sequenced transcriptome. Among these unigenes, 38.61% (56,964) were successfully annotated in at least one database, and 2.78% (4,106) were annotated across all databases. Annotation rates varied among databases: the NR database showed the highest annotation rate (27.96%), followed by PFAM and GO (both 23.98%), SwissProt (20.85%), and NT, KO and KOG (14.12%, 11.65%, and 7.12%, respectively).

**Figure 7:**
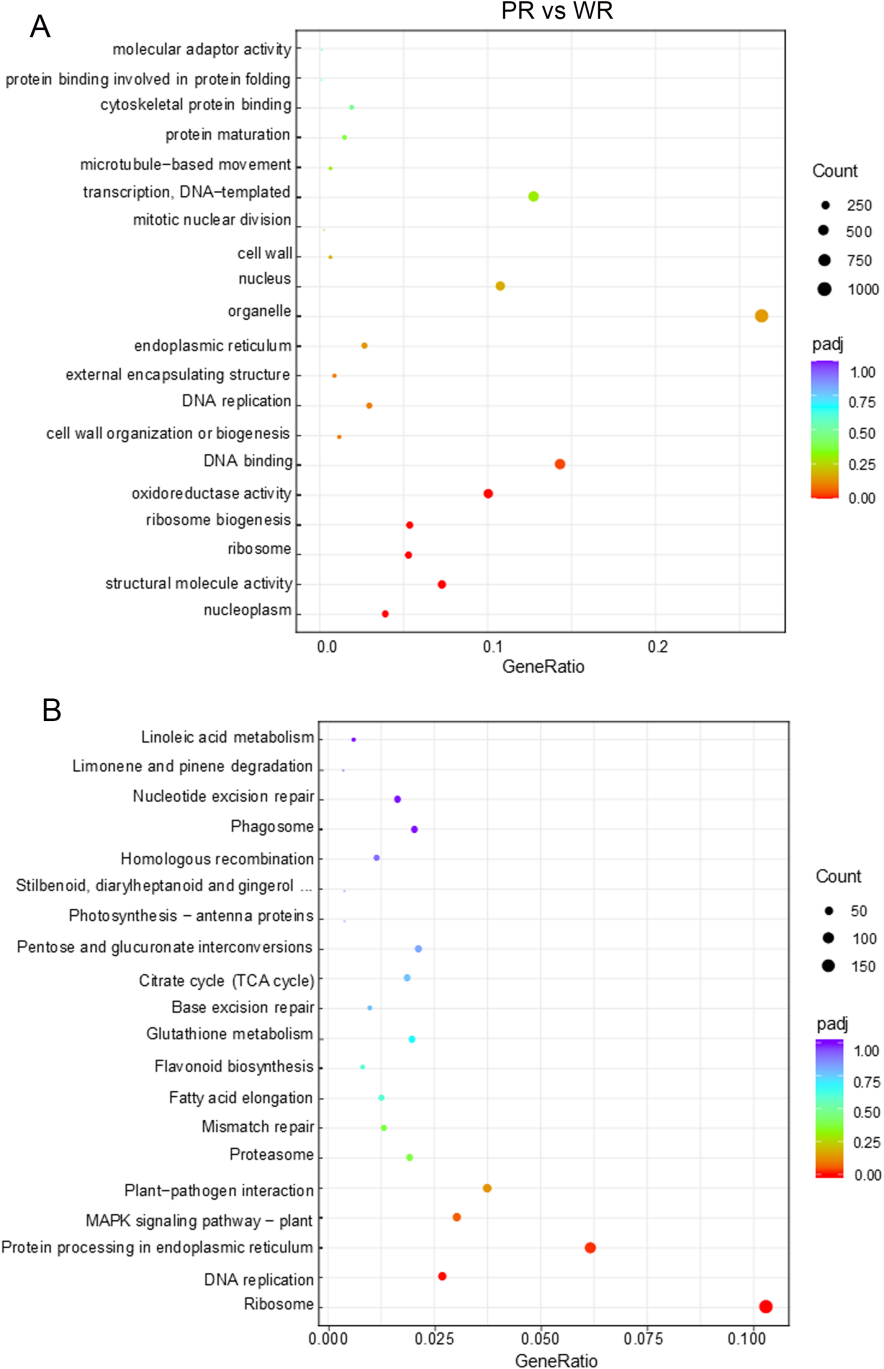
Functional enrichment analysis results of the comparative transcriptome between PR and WR in Chinese chive. **(A)** Bubble plot of GO functional enrichment analysis. **(B)** Bubble plot of KEGG pathway enrichment analysis.

KEGG pathway enrichment and GO functional enrichment analyses were performed on the identified DEGs to clarify their biological functions. KEGG pathway enrichment results revealed that DEGs were predominantly enriched in core cellular metabolic and regulatory pathways. Among these pathways, the ribosome pathway was the most significant enrichment and contained the largest number of DEGs, indicating a marked difference in protein synthesis between the two groups (**Figure 7B**). In addition, protein processing in endoplasmic reticulum and DNA replication pathways were also significantly enriched, suggesting distinct differences in protein maturation, folding, and DNA replication/cell proliferation between PR and WR groups. Pathways related to environmental response, such as the plant MAPK signaling pathway and plant-pathogen interaction, showed marginally significant enrichment. This implied potential differences in stress adaptation and immune regulation between the two groups. GO functional enrichment analysis further corroborated these findings (**Figure 7A**). At the Molecular Function level, terms including structural molecule activity, ribosome biogenesis, and ribosome were significantly enriched, which was consistent with KEGG results and highlighting dysregulation of ribosomal function and protein synthesis. Meanwhile, terms such as oxidoreductase activity and DNA binding were also significantly enriched, indicating alterations in oxidative stress responses and transcriptional regulation between the two groups. At the Cellular Component level, DEGs were enriched in organelles, the nucleus, and the endoplasmic reticulum—primary subcellular locations for protein synthesis, processing, and DNA replication—further corroborating the conclusions of the KEGG pathway analysis.

Integrating the above results, a key SSR marker sxauAt427 significantly associated with anthocyanin content was identified in the transcriptomic analysis between PR and WR Chinese chives. This marker showed significant co-association with the expression pattern of Unigene-84067, which encodes hydroxycinnamoyltransferase (HCT) involved in the flavonoid biosynthesis pathway. Annotations from KEGG and SwissProt databases confirmed that Unigene-84067 encodes shikimate O-hydroxycinnamoyltransferase (HCT), which functions upstream of the flavonoid and anthocyanin biosynthesis pathways. As a core precursor-supplying enzyme, it provides essential precursors for the biosynthesis of flavonoids including anthocyanins, representing one of the important molecular mechanisms underlying the difference in total anthocyanin content between PR ang WR Chinese chives.

**Figure 8:**
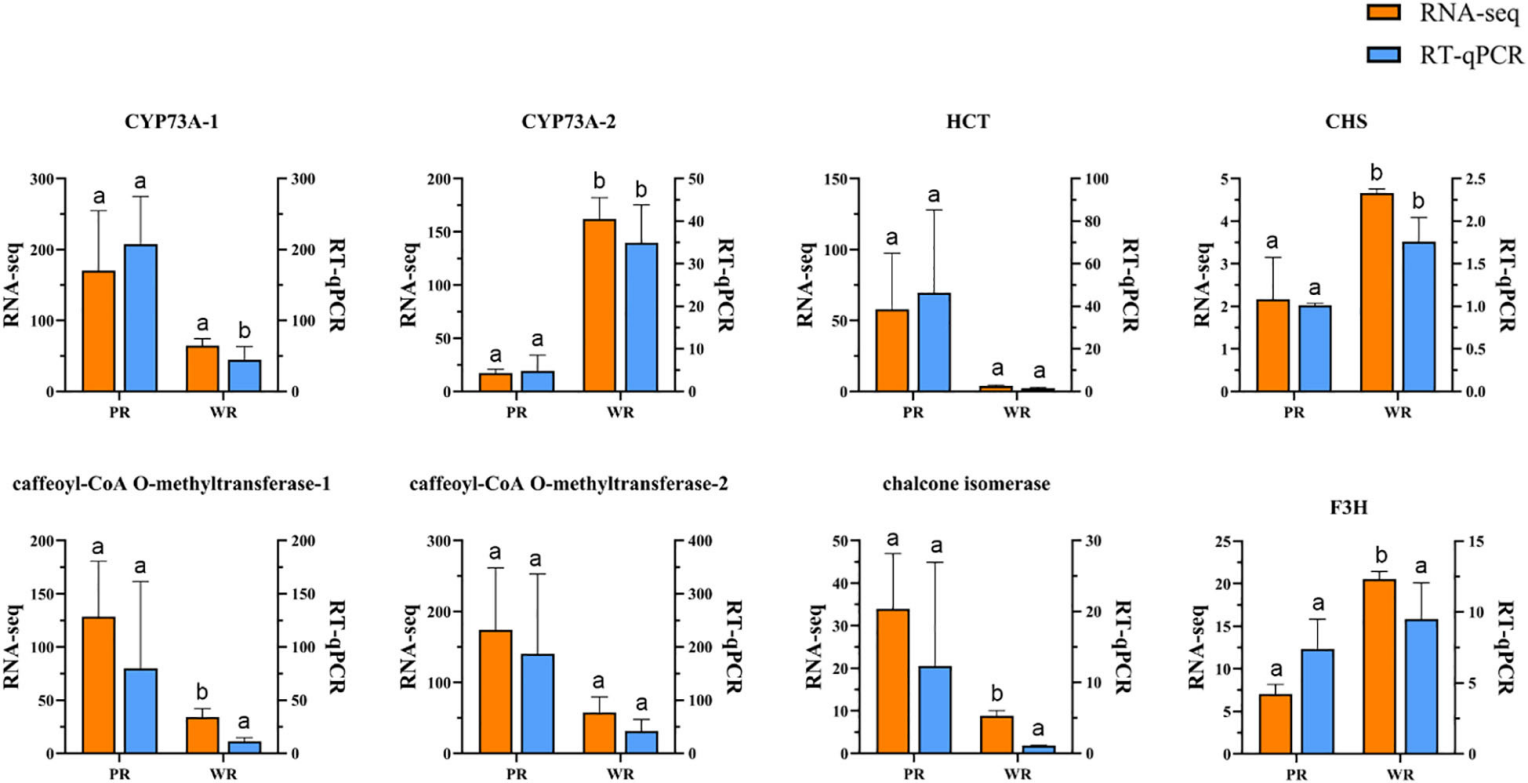
RT-qPCR validation results of relative expression levels of 8 Anthocyanin pathway genes (RNA-seq expression values are presented as FPKM).

RT-qPCR results demonstrated that the expression patterns of these eight genes were remarkably consistent with those derived from RNA-Seq, thereby confirming the robustness of the transcriptomic findings (**Figure 8**).

**Figure 9:**
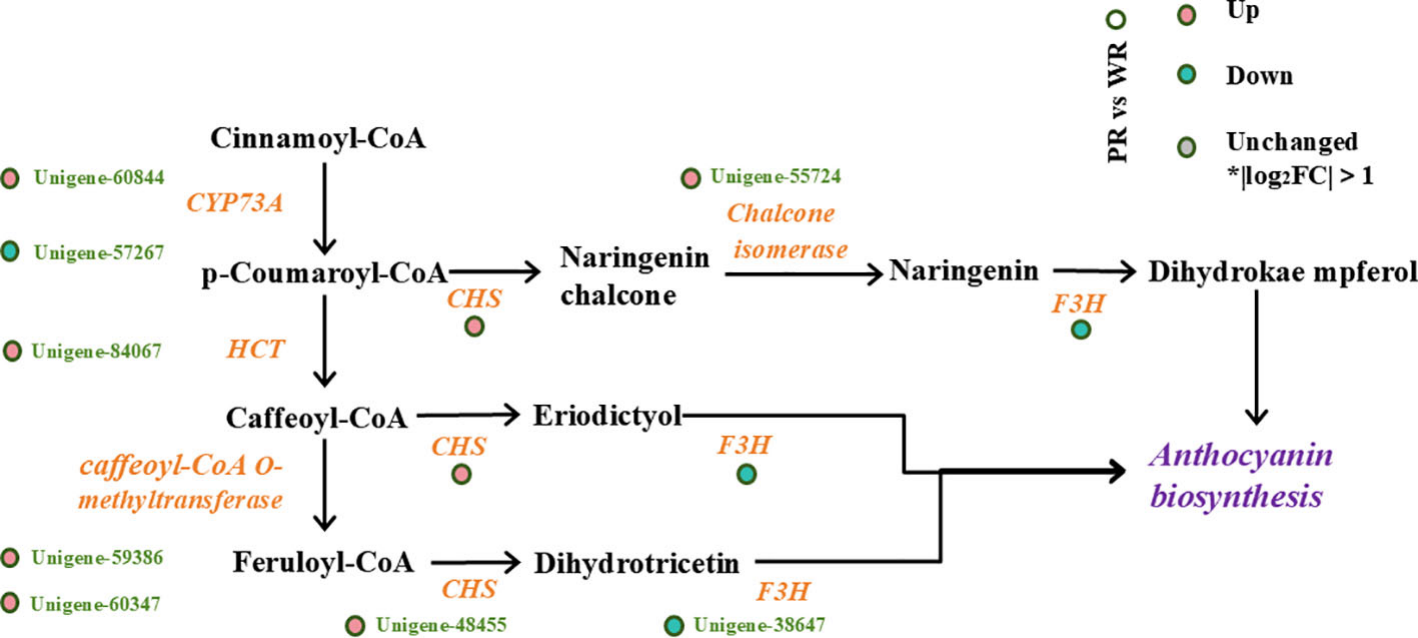
Anthocyanin biosynthesis-related pathway and gene differential expression map involving DEGs associated with Unigene-84067(HCT) between PR and WR.

Furthermore, we constructed a pathway map of DEGs associated with the expression pattern of Unigene-84067 to clarify the anthocyanin biosynthesis process. This pathway starts with cinnamoyl-CoA, a product of phenylpropanoid metabolism, which is catalyzed by CYP73A to form p-coumaroyl-CoA. Subsequently, p-coumaroyl-CoA is converted by HCT into intermediates including caffeoyl-CoA and feruloyl-CoA. These intermediates enter different branches of flavonoid biosynthesis and ultimately converge into the anthocyanin biosynthesis pathway to yield various anthocyanin products. (**Figure 9**).

## 4 Discussion

Agronomic trait investigation is the core link connecting plant genetic information with field performance. It serves as both an important foundation for modern agricultural research and a key basis for guiding production practices. Compared with molecular marker technology, germplasm resource evaluation and identification based on agronomic traits have the advantages of intuitiveness, high efficiency, and simple operation. Accurate agronomic trait investigation can directly provide support the excavation of excellent traits, germplasm innovation, and the formulation of breeding objectives (Yu et al., 2006). In this study, 18 important agronomic traits of Chinese chives were investigated to lay a foundation for subsequent breeding work. Principal component analysis (PCA) results showed that the core indicators of the first principal component (PC1) included growth-related traits such as PH, LW and PD. This finding is highly consistent with the conclusions of previous studies on Chinese chive agronomic traits (Li et al., 2020b). It further confirms at the genetic level that these traits are regulated by a common genetic regulatory mechanism and collectively characterize the overall vegetative growth potential of plants. This provides a theoretical basis for dissecting the genetic regulatory network of Chinese chive growth and development.

However, it should be noted that most agronomic traits are quantitative traits. Traditional conventional breeding techniques often face limitations in directional improvement, such as limited improvement effects, long breeding cycles, and low selection efficiency (Dai et al., 2025). Therefore, using molecular markers for early selection of agronomic traits and marker-assisted breeding has become an important development direction in genetic improvement. This also serves as the core starting point for conducting SSR marker association analysis in this study.

This study adopted a “public transcriptome dataset + specific transcriptome dataset” strategy to develop SSR markers. The public transcriptome dataset (PRJNA673978) enabled the rapid acquisition of a large number of candidate loci, effectively making up for the deficiency of the lack of a complete reference genome for Chinese chive. Aligning this public dataset with the species-specific transcriptome of this study allowed for the accurate screening of markers matching the genetic background of the tested materials, greatly improving experimental reliability. This strategy conforms to the conventional thinking of molecular marker development and is highly adapted to the unique genetic characteristics of Chinese chive, such as autotetraploidy and an ultra-large genome. Notably, all markers used in this study were EST-SSRs, which are directly derived from functional transcripts and facilitate the “marker-trait-gene” linkage analysis. Nevertheless, EST-SSRs have inherent biases. First, they are biased towards gene coding regions and fail to cover non-coding regions, potentially missing key associated loci. Second, their polymorphism depends on gene expression, making it difficult to capture genetic variations in low/non-expressed regions. These biases have to a certain extent limited the number of markers and genome coverage, and also pointed out the optimization direction for subsequent marker development work.

Fifty-nine SSR markers were selected for the association analysis of autotetraploid Chinese chive, and this selection was determined by the unique genomic characteristics of Chinese chive.

Chinese chives are autotetraploids (2n=4×=32) without a chromosome-level reference genome. Their genome is approximately 31.2 Gb with an extremely high proportion of repetitive sequences (Zhou et al., 2017; Zhang et al., 2025). These characteristics make SSR markers prone to non-specific amplification, blurred bands, multiple peaks and other problems, which hinder their application in population genotyping (Tang et al., 2017). To address this issue, this study conducted multiple rounds of rigorous screening of 500 SSR markers developed in the early stage. Finally, 59 core markers with stable amplification, clear bands, and moderate polymorphism were obtained. These markers can effectively distinguish the genetic differences of the tested population and ensure the reliability of association analysis.

Population structure analysis is a prerequisite and foundation for association analysis. Complex population stratification is prone to causing false positive association results (Huang et al., 2025), while a clear and stable population structure helps improve the reliability of association analysis (Liu et al., 2022). In this study, both UPGMA cluster analysis and Structure population structure analysis divided 82 Chinese chives into 3 core groups. The classification results were highly consistent for most germplasms, with only 11 germplasms showing differences. Combined with phenotypic and genetic characteristics, the breeding value of the three groups can be clearly defined. Pop I exhibits a large variation range of plant morphological phenotypes, significant individual differences, and diverse genetic backgrounds, making it suitable as a basic resource for gene mining and association analysis. Pop II has relatively uniform phenotypes and contains specific germplasms with extremely long flower stalks and high anthocyanin content, which are key materials for quality improvement. Pop III features vigorous vegetative growth and strong bolting ability, and can be used as core parents for high-yield breeding.

Association analysis combined with molecular marker-assisted breeding has become an efficient approach for crop breeding. Its feasibility has been confirmed by relevant studies on cotton, pepper, tartary buckwheat and other crops (Siddho et al., 2025; Duan et al., 2025; Hou et al., 2022), providing a solid theoretical reference for this study. In this study, the general linear model (GLM) and mixed linear model (MLM) with Q and Q+K as covariates were used for association analysis. This approach was adopted to control the false positive risk caused by population stratification and genetic relationships, thereby improving the reliability of results (Ashfaq et al., 2022; Cho et al., 2024). The results showed that the MLM detected 30 significantly associated markers, far more than the 2 markers identified by the GLM. This indicates that the MLM can effectively correct the influence of genetic relationship and more accurately capture the association signals of complex traits controlled by multiple genes. It also reflects the complexity of the genetic regulatory mechanism of agronomic traits in Chinese chive. The innovation of this study lies in the first systematic association of EST-SSR markers with 18 agronomic traits of Chinese chive. The 59 core markers obtained can serve as a direct technical tool for molecular marker-assisted breeding of Chinese chive.

Among the association results, sxauAt427 has the most prominent biological significance: this marker was significantly associated with AC in both models and was also significantly correlated with Unigene-84067. Unigene-84067 is a gene in the flavonoid synthesis pathway encoding hydroxycinnamoyl-CoA shikimate/quinate hydroxycinnamoyl transferase (HCT). As a key enzyme in the phenylpropanoid pathway, HCT acts upstream in the anthocyanin biosynthesis pathway and provides key precursors for this process. This finding reveals the molecular regulatory basis for differences in anthocyanin content of Chinese chives. Previous studies have shown that HCT plays a conserved regulatory role in anthocyanin synthesis in *Rhododendron, Platycodon grandiflorum, Solanum tuberosum*, and other species (Zhu et al., 2016; Nie et al., 2019; Li et al., 2022b; Qi et al., 2025; Wang et al., 2025). This further supports the reliability of the results of this study and indicates that sxauAt427 has the potential to be developed as a functional marker for anthocyanin traits.

In addition to sxauAt427, this study also screened a number of SSR markers closely associated with important agronomic traits. Among them, sxauAt343 was specifically associated with LW and can be directly used for the early directional screening of broad-leaved and high-yield Chinese chive germplasms. sxauAt186 was simultaneously associated with LP and LA, enabling synchronous selection of two yield-related plant type traits with a single marker and greatly improving breeding screening efficiency. Furthermore, sxauAt068 was associated with LP, LC, and FSS, while sxauAt125 and sxauAt372 exhibited multi-effect associations with LA, FSS, NL and FSS, respectively. These SSR markers, which have both specificity and pleiotropy, complement each other. They can accurately support the screening and improvement of high-yield, high-quality and characteristic Chinese chive germplasms, and provide core available molecular tools for the molecular marker-assisted breeding of Chinese chive agronomic traits.

It should be objectively pointed out that these markers have only been verified based on single-environment data. Their environmental universality and adaptability to different genetic backgrounds need to be further verified. In future studies, multi-environment and multi-genetic background field experiments should be carried out to eliminate the influence of genotype × environment interaction (G×E). Meanwhile, more trait-associated markers and functional verification should be supplemented to give full play to the breeding application value of these markers in cultivating high-yield, high-quality, and characteristic Chinese chive varieties.

This study still has some deficiencies in research design and verification depth, which need to be improved in subsequent work. The association between sxauAt427 and the HCT gene was only obtained based on transcriptome co-expression analysis and marker-trait association analysis. The true genetic linkage relationship between them has not been verified by physical mapping, making it impossible to clarify their specific positional relationship on chromosomes. Only 3 plants of each germplasm were observed in phenotypic determination, which limited the statistical power of phenotypic data. In addition, the single-environment experimental design cannot effectively analyze the influence of G×E on trait expression, making it difficult to evaluate the environmental stability of marker-trait associations. The HCT gene related to anthocyanin synthesis has only completed association analysis so far, and lacks systematic functional verification experiments such as gene silencing and overexpression. Thus, its specific regulatory mechanism and regulatory network in Chinese chive anthocyanin synthesis remain unclear.

To address the above deficiencies, targeted supplementary and optimization work will be carried out in subsequent studies. First, the genetic linkage relationship between sxauAt427 and the HCT gene will be verified through fine genetic mapping and physical mapping to clarify their chromosomal localization information. Second, the phenotypic identification scheme will be optimized by increasing the number of observed plants per germplasm to improve statistical power. Multi-year and multi-site field experiments will be set up to systematically analyze the influence of G×E on the expression of agronomic traits of Chinese chive and evaluate the environmental stability of core markers. Third, functional verification of the HCT gene will be conducted through molecular biological methods such as gene silencing and overexpression, combined with metabolome analysis to clarify its specific regulatory mechanism and action sites in Chinese chive anthocyanin synthesis. Meanwhile, this study will continue to develop more molecular markers with wider coverage and stronger applicability relying on GBS technology and pan-genome construction, providing more abundant tool support for Chinese chive molecular breeding.

In summary, through the combined analysis of phenotypic identification, population structure analysis, EST-SSR marker association analysis and transcriptome data, this study successfully screened a set of SSR markers that can be directly applied to Chinese chive molecular marker-assisted breeding. It also initially revealed the genetic regulatory basis of major agronomic traits of Chinese chive agronomic traits, especially the anthocyanin content trait. Additionally, an objective analysis and discussion on the methodological biases of EST-SSR markers were conducted. The results of this study can provide important theoretical reference and technical support for the innovation of Chinese chive germplasm resource innovation and molecular marker-assisted breeding. They also have important theoretical and practical significance for promoting research on the genetic mechanisms and excellent variety improvement of Allium crops.

## 5 Conclusion

In this study, the biologically meaningful marker sxauAt427 that is significantly associated with anthocyanin content was identified. The dataset constructed in this study can provide data support for future genetic dissection, candidate gene screening, functional validation, and fine-mapping of relevant traits in Chinese chive. The identified marker exhibits potential for molecular marker-assisted breeding, yet its breeding value still requires further validation through independent population testing, multi-environment evaluation and fine mapping. The results of this study can serve as a reference for the germplasm resource development and molecular breeding of Chinese chives.

## Data Availability

The data presented in the study are deposited in the National Genomics Data Center (NGDC), accession number CRA039881.
