# Supplementary material for: Genetic diversity and association analysis between agronomic traits and EST-SSR markers in Chinese chive (Allium tuberosum)
Source: Front Plant Sci. 2026 Mar 23;17:1785981. doi: 10.3389/fpls.2026.1785981 (PMC13050861; doi:10.3389/fpls.2026.1785981)
Supplement: Supplementary file 1 [file Presentation1.pptx]

## Slide 1
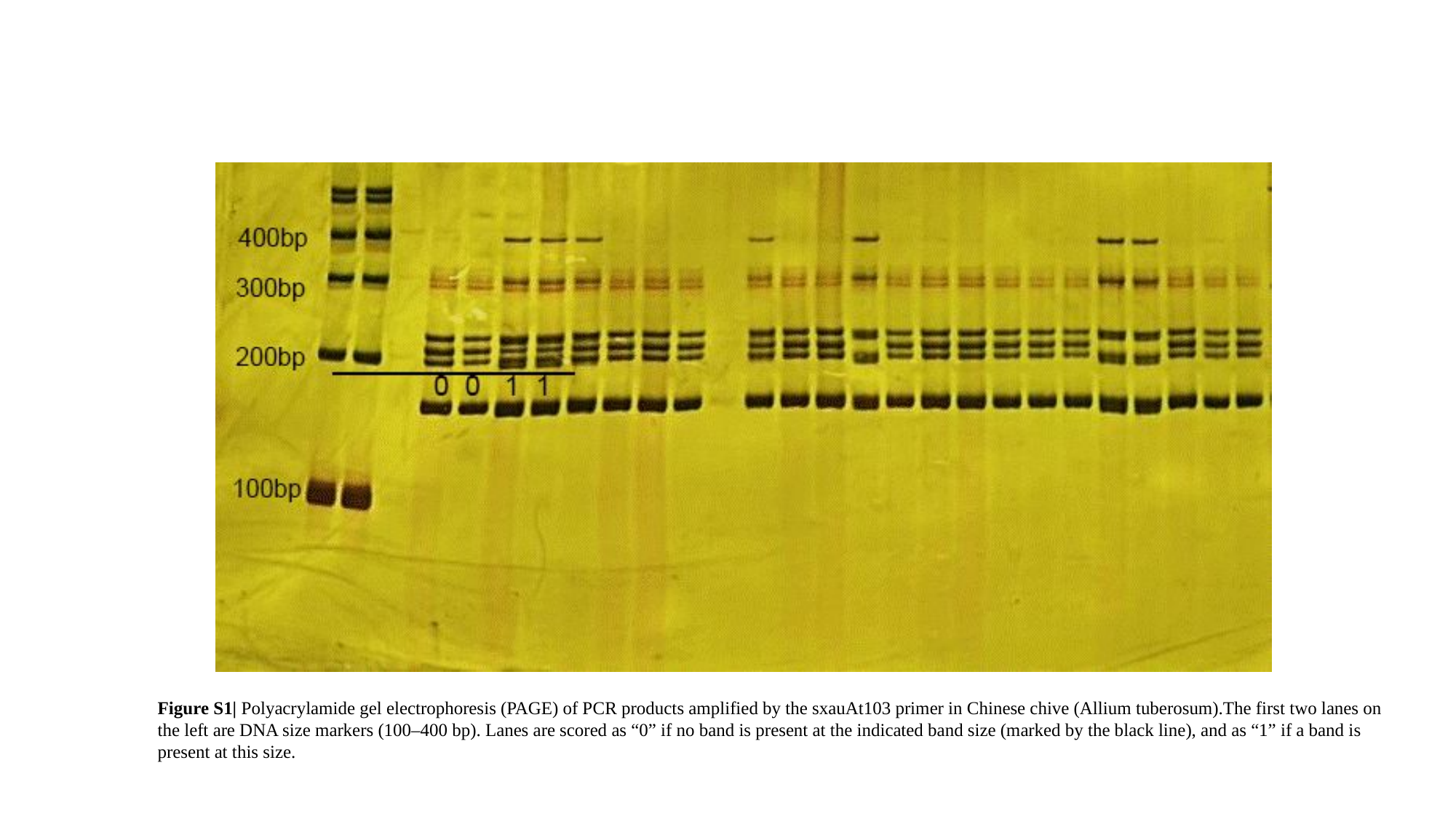

Figure S1| Polyacrylamide gel electrophoresis (PAGE) of PCR products amplified by the sxauAt103 primer in Chinese chive (Allium tuberosum).The first two lanes on the left are DNA size markers (100–400 bp). Lanes are scored as “0” if no band is present at the indicated band size (marked by the black line), and as “1” if a band is present at this size.

## Slide 2
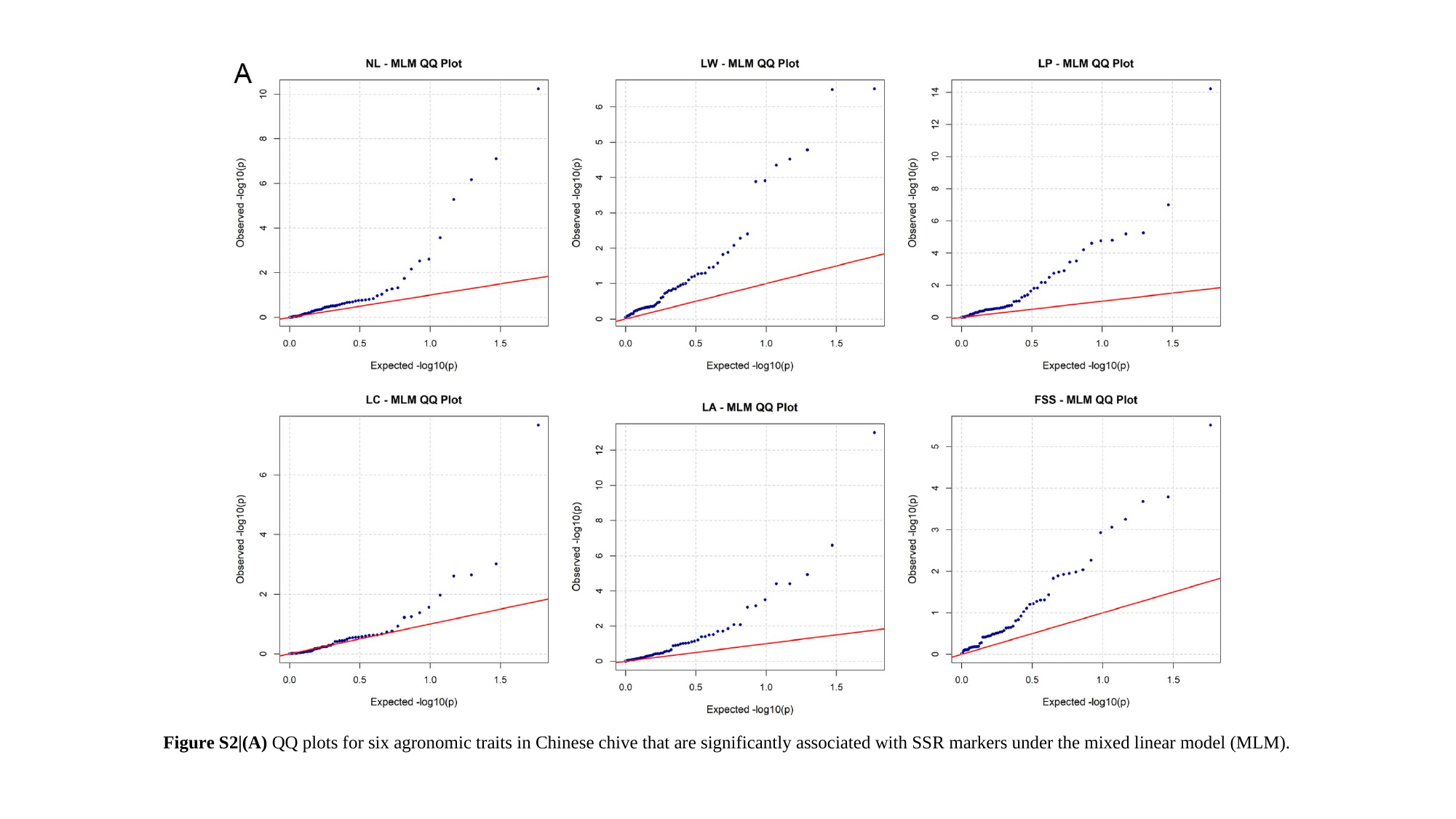

Figure S2|(A) QQ plots for six agronomic traits in Chinese chive that are significantly associated with SSR markers under the mixed linear model (MLM).

## Slide 3
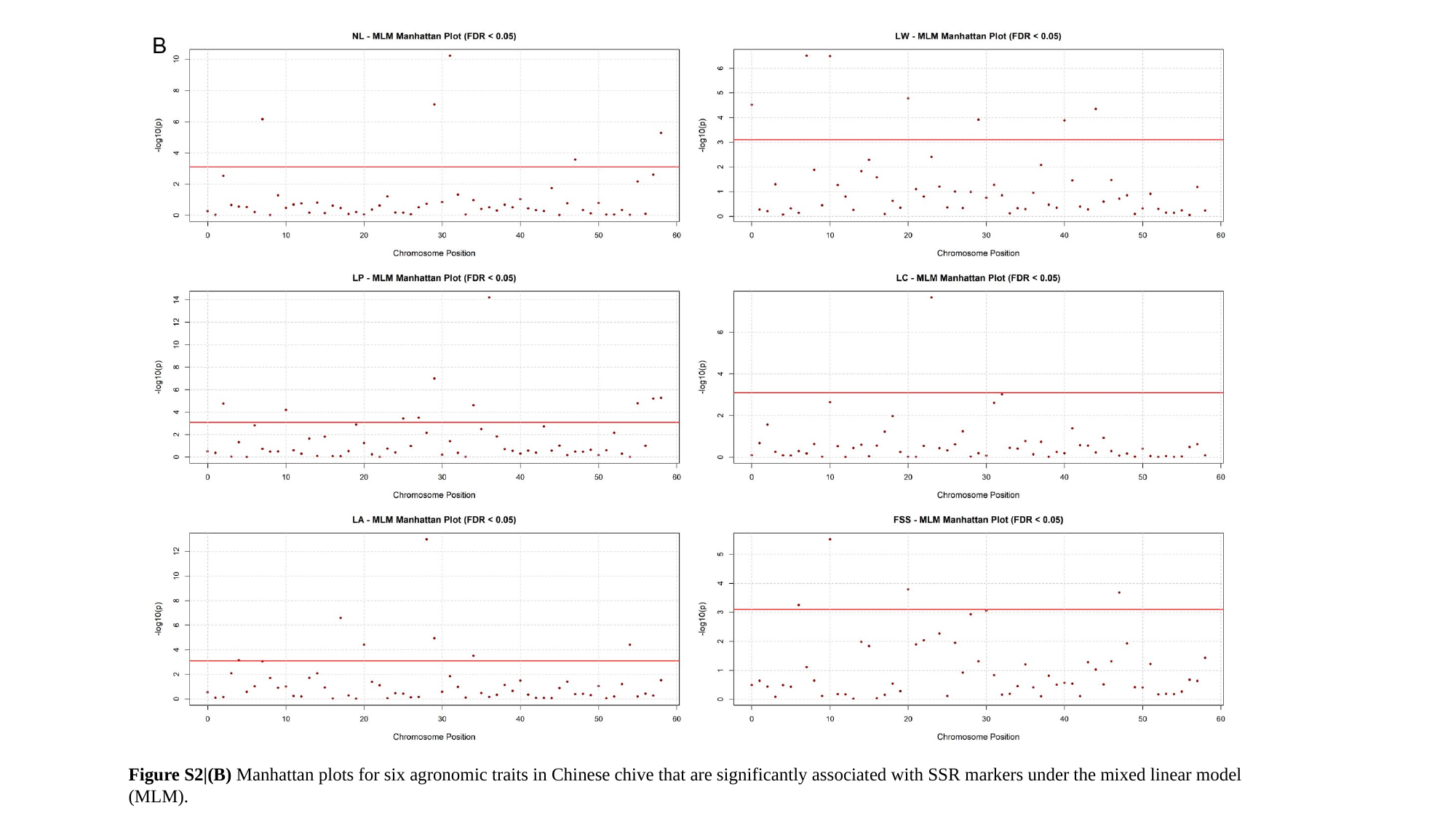

Figure S2|(B) Manhattan plots for six agronomic traits in Chinese chive that are significantly associated with SSR markers under the mixed linear model (MLM).

## Slide 4
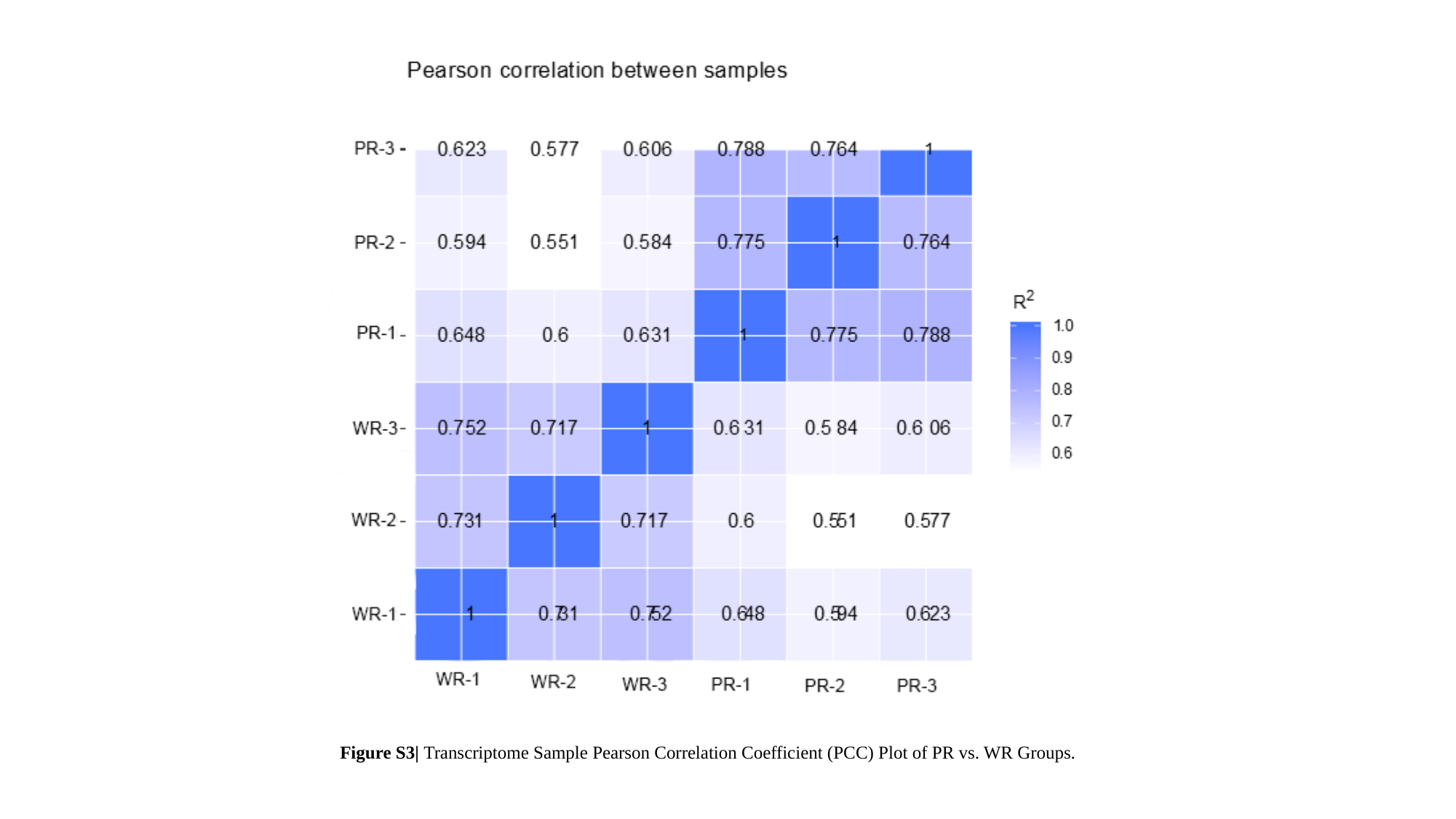

Figure S3| Transcriptome Sample Pearson Correlation Coefficient (PCC) Plot of PR vs. WR Groups.

## Slide 5
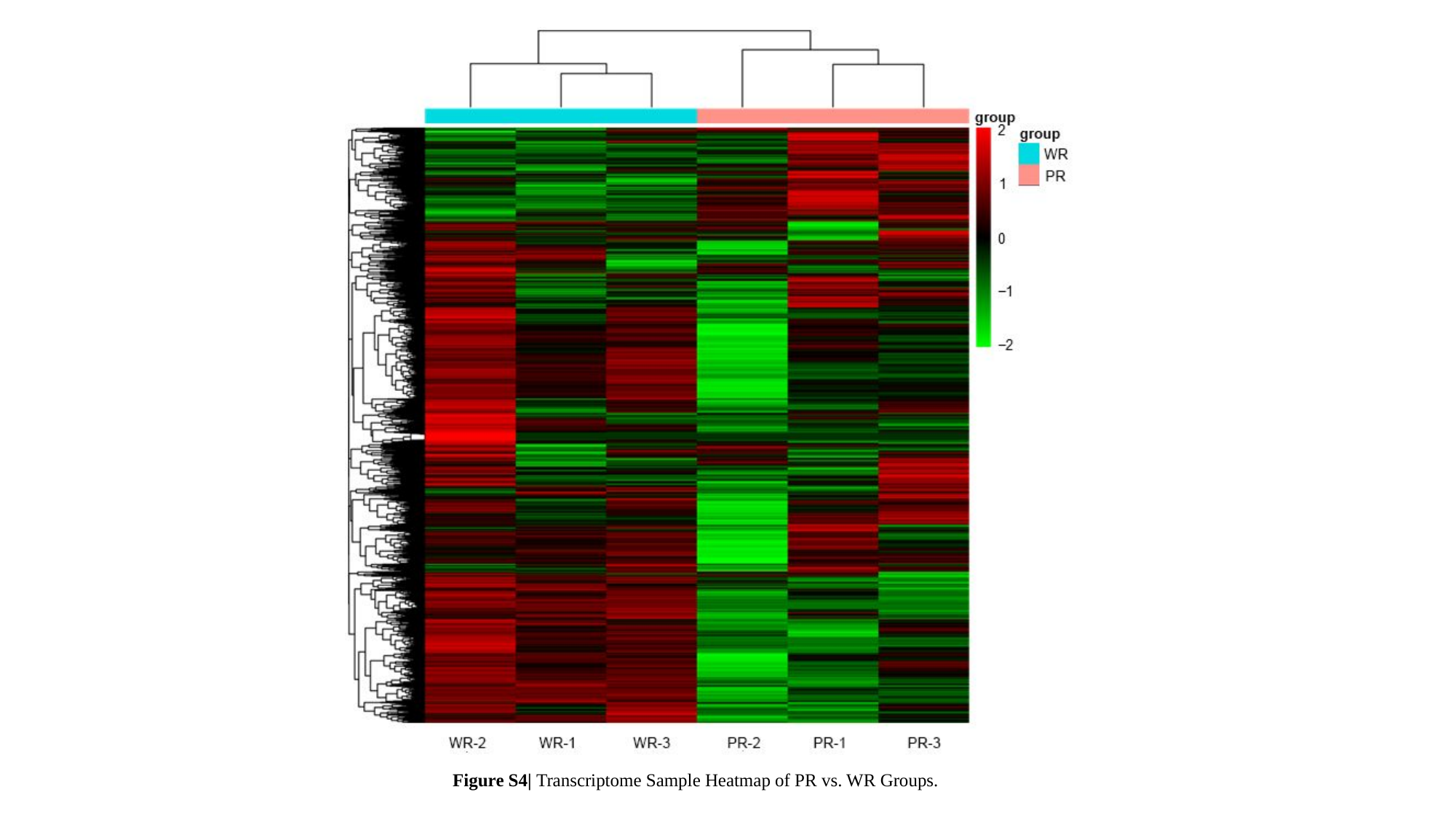

Figure S4| Transcriptome Sample Heatmap of PR vs. WR Groups.

## Slide 6
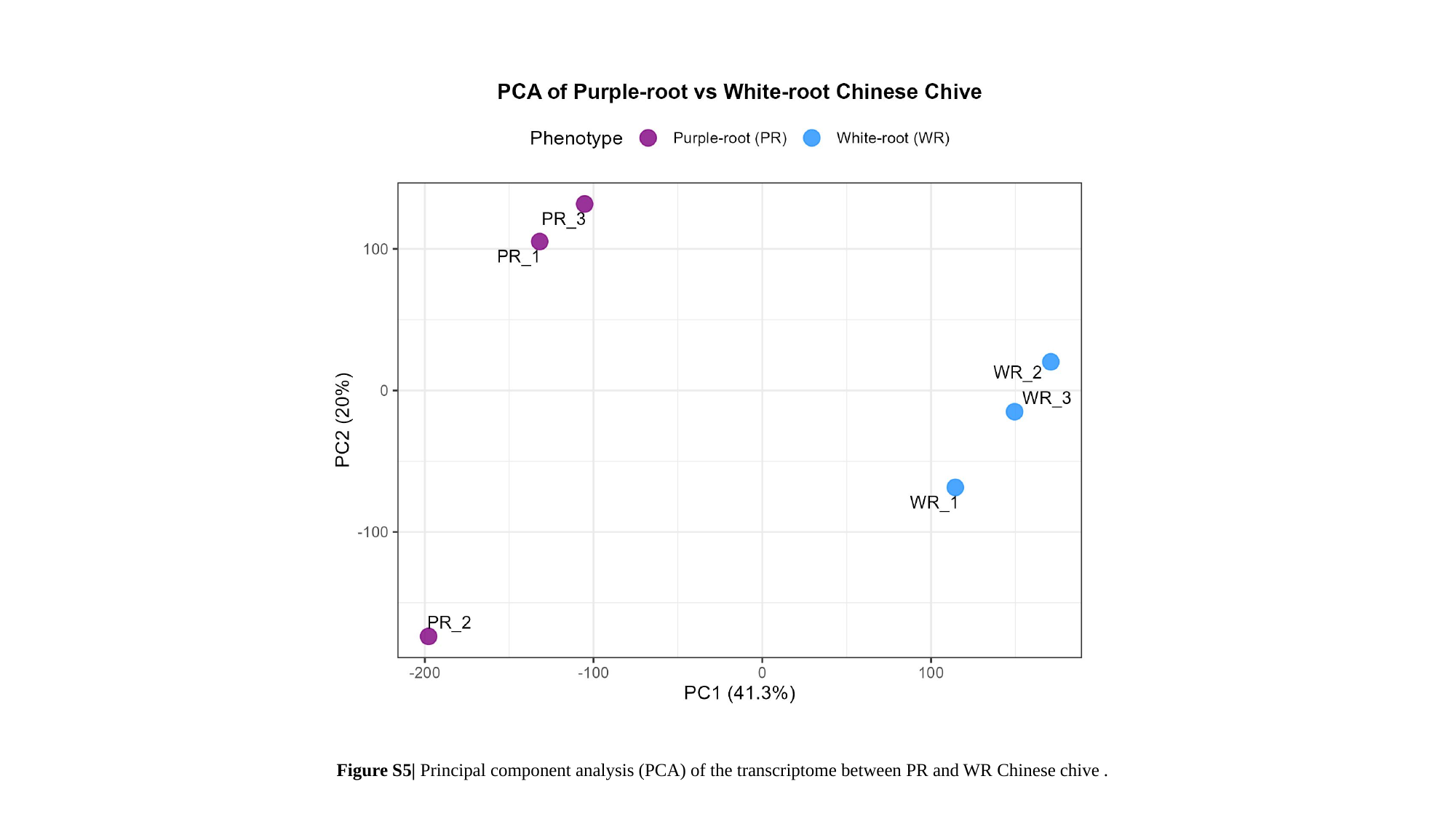

Figure S5| Principal component analysis (PCA) of the transcriptome between PR and WR Chinese chive .
